# Analysis of Cationic Vitamins in Cell Culture Medium Samples by Capillary Zone Electrophoresis

**DOI:** 10.1155/2022/2819855

**Published:** 2022-10-06

**Authors:** Debbie van der Burg, Hermann Wätzig, Cari E. Sänger–van de Griend

**Affiliations:** ^1^Kantisto BV, Callenburglaan 22, 3742 MV, Baarn, Netherlands; ^2^KTH Royal Institute of Technology, Department of Chemistry, Division of Applied Physical Chemistry, Stockholm, Sweden; ^3^Institute of Medicinal and Pharmaceutical Chemistry, TU Braunschweig, Braunschweig, Germany

## Abstract

This paper describes a capillary electrophoresis method for the determination of the cationic B-vitamins thiamine, nicotinamide, pyridoxine, pyridoxal, and pyridoxamine in untreated cell culture medium samples. The effects of the buffering capacity, the mobility of the coion, and the preconditioning solution on the robustness of the method were investigated. Using a 100 mM phosphoric acid and 55 mM triethanolamine background electrolyte at pH 2.3 and capillary preconditioning with 1 M NaOH, all five vitamins could be separated with good resolution. Preliminary method validation data over the range 10–110 *µ*M for undiluted samples, with 10 *μ*M being the lower range limit of quantification QL, showed accuracy recoveries of 94–104%, and migration time and peak area repeatabilities within 0.4% RSD and 2.6% RSD, respectively.

## 1. Introduction

Biopharmaceuticals have become important products in the pharmaceutical industry [1, 2]. Biopharmaceuticals are produced using cell culture bioprocesses. The cell culture medium quality is critical for bioprocess performance and the quality of the final product [3]. Vitamins are present in cell culture medium in small amounts as essential nutrients. They have been shown to play important roles in cell growth, cell death, and productivity [4, 5], as well as for an increase in mAb yield [6]. Vitamins can also affect the colour of the drug substance, which is a product quality attribute [6, 7]. For a better understanding of the effect of vitamins on factors such as cell growth, cell viability, and productivity, the monitoring of the biopharmaceutical process is essential. The degradation of vitamins in aqueous solutions can make accurate analysis challenging. B-vitamins are sensitive to a range of external factors, such as light, oxygen, low or high pH, and temperature, as well as to interactions with other cell culture medium components [8]. Vitamin monitoring is nowadays often performed offline, where samples are taken at different time points of the biopharmaceutical process and stored for analysis at a later time point. Due to the unstable character of the vitamins in solutions, there is a need for a fast, robust method for cell culture medium samples that can be used for at-line analysis directly after sampling. A separation technique that can handle the complex cell culture medium, consisting of the medium components including carbohydrates, amino acids, vitamins, lipids, salts, trace elements, growth factors, polyamines, buffers, surfactants, and antifoams [9], as well as components produced or leaked by the cells, such as metabolites, proteins, nucleic acids, lipids, and membrane debris, should be selected. In addition to the complex sample matrix, vitamins, even though they are all essential nutrients, show few close chemicals or functional similarities [10]. Due to the different physical-chemical properties, a one-size-fits-all derivatisation or sample clean-up is difficult. Capillary electrophoresis (CE) can handle complex sample matrices with reduced sample preparation because of its high separation power and simple set-up. In addition, with CE, rapid analyses can be obtained with minimal sample volumes. For at-line monitoring of the biopharmaceutical process, a robust method should be developed. Several factors impact migration time and peak area repeatability. Controlling the electro-osmotic flow (EOF) is one of the most important factors for robustness and continuous reproducible use. The EOF is the result of the electric double layer formed by ions in the buffer to balance the charge on the capillary wall. When an electric field is applied over the capillary, ions in the diffuse part of the electric double layer move and drag the bulk liquid along, creating a plug flow. The EOF is affected by several factors, such as the ionic strength of the buffer and the charge on the capillary wall [11]. The charge of bare fused silica capillary walls is pH dependent. The charge on the capillary wall can be minimised by selecting a background electrolyte (BGE) with a low pH, this significantly reduces, and thus controls, the EOF. In addition, the minimised charge reduces the adsorption of matrix components to the capillary wall. Adsorption of matrix components to the capillary wall could affect the charge on the wall and thus the EOF. Adsorption of cell culture medium components cannot be fully avoided, so in order to further reduce the adsorption of components from the complex cell culture medium samples, capillary coatings could be applied. Next to preventing adsorption, capillary coatings also control the EOF. One option is dynamically coating the capillary by adding an additive to the BGE, such as amine modifiers like triethanolamine (T-EthA). T-EthA could be used to suppress adsorption because of its electrostatic interaction with silanol, masking free silanol groups [12–14]. These BGE additives can form a positively charged layer on the capillary wall, protecting it from the adsorption of cell culture medium components, and slowing down or even reversing the EOF. The applied voltage during separation can cause electrolysis of the BGE, altering its pH. Since the pH both affects the EOF and the analyte charge, the pH of the BGE should be kept constant. Buffer depletion could be reduced by selecting a BGE with a high buffering capacity [15]. Several factors influence the buffering capacity, such as the concentration and the pH of the BGE; a higher buffering capacity is obtained with a higher buffer concentration and when a buffer pH close to the pKa of the buffering component is selected. By selecting the BGE pH, it is also important to take the pKas of the analytes into account, for a robust method, the charge on the analytes should not be significantly affected by small pH changes; and thus, a pH significantly far from the analytes pKas should be selected. Lastly, capillary preconditioning is important for high migration time repeatabilities [16, 17]. Rinsing the capillary is important since it returns the capillary to the same consistent conditions. The capillary should first be rinsed with a solution to remove potentially adsorbed components from the capillary wall, usually, sodium hydroxide or strong acids in the range of 0.1 to 1.0 M are used. Then the capillary should be rinsed with the BGE to re-equilibrate the capillary surface. Good capillary preconditioning can provide a well-defined, reproducible state of surface hydroxylation on the capillary wall. Although several papers describing CE for the analysis of water-soluble vitamins in pharmaceutical preparations such as tablets were published [18–22], the monitoring of vitamins with CE in biopharmaceutical cultivation processes remained unexplored. Some interesting work has been shown for vitamin analysis in the bacterial growth medium [23]. However, the BGE in this work has a high conductivity combined with a low buffering capacity. The presence of sodium dodecyl sulphate in the BGE can potentially prevent the adsorption of medium components to the capillary wall, but seeing the complex nature of upstream cell medium samples, this is likely not sufficient. For pharmaceutical tablets, often only pyridoxine is used as B6 vitamin, whereas in cell culture cultivation processes, a variation of all three B6 vitamins, pyridoxine, pyridoxal, and pyridoxamine is used. In this work, the focus is on developing a method for the detection of the cationic B-vitamins thiamine, nicotinamide, pyridoxine, pyridoxal, and pyridoxamine in upstream processing cell culture medium samples with CE. During method development, factors affecting the robustness, such as capillary preconditioning, buffering capacity, buffer pH, and coion mobility were investigated.

## 2. Experimental

### 2.1. Chemicals

Thiamine HCl, nicotinamide, pyridoxine HCl, pyridoxal HCl, pyridoxamine 2HCl, Tris(hydroxymethyl)aminomethane (Tris), phosphoric acid 85–95%, glycine, and triethanolamine (T-EthA) were obtained from Merck/Sigma Aldrich (Darmstadt, Germany). HyClone ActiPro medium and HyClone ActiSM medium were purchased from Cytiva (Marlborough, USA). Modified FMX-8 cell culture medium (FMX-8 MOD) with the composition as described in [24] was provided by the Department of Industrial Biotechnology, KTH Royal Institute of Technology, Stockholm, Sweden. Water was of MilliQ-grade quality (18.2 MΩ·cm).

### 2.2. Instrumental Conditions

Experiments were conducted on an Agilent 7100 capillary electrophoresis system with a Diode Array UV detector (Waldbronn, Germany). Chemstation software was used for instrument control, data acquisition, and data analysis. Bare fused silica capillaries with 50 *µ*m id were purchased from Agilent Technologies. Capillaries had a total length of 33 cm with an effective length of 24.5 cm. The separation voltage ranged from 13 kV to 20 kV depending on the conductivity of the used (BGE) and was ramped over 0.5 min. Samples were introduced hydrodynamically at 30 mbar for 5 s, followed by the injection of a BGE plug using the same conditions. Separations were carried out at 20 °C. The detector signal was recorded at 210 nm. Before first use, the capillary was flushed successively with 1 M NaOH, water, and BGE at 1 bar for 20 min each. At the beginning of each working day, the capillary was flushed successively with 0.1 M NaOH, water, and BGE at 1 bar for 10 min each. Prior to each injection, the capillary was preconditioned with BGE at 1 bar for two minutes.

### 2.3. BGE and Sample Preparation

BGEs containing phosphoric acid under acidic conditions were tested with different coions; Tris, glycine, and T-EthA. The final BGE consisted of 100 mM phosphoric acid and 55 mM T-EthA, which had a pH of 2.3. No pH adjustment was done. Vitamin stock solutions of 20 mM in water were prepared and stored at −20 °C. A standard vitamin mixture was prepared by mixing these vitamin stock solutions and diluting the mixture with water, the final concentrations of the vitamins in the mixtures varied and ranged from 10 *µ*M to 110 *µ*M. Cell culture medium samples were analysed untreated. Spiked cell culture medium samples were prepared by adding 4 *µ*L of a 4.5 mM standard vitamin mixture to 356 *µ*L cell culture medium sample. Descriptive statistics were calculated according to the normal procedures.

## 3. Results and Discussion

### 3.1. Method Development

**Figure 1:**
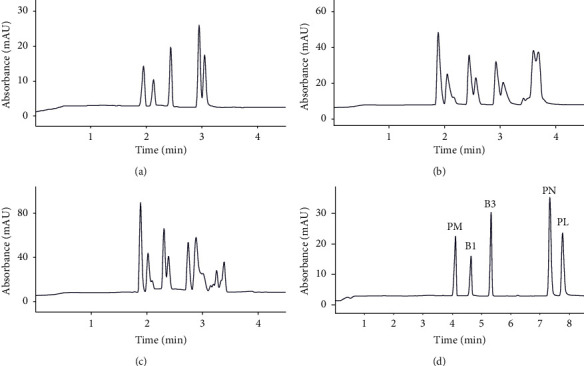
Electropherograms of a standard vitamin mixture (conc. 2.5–4.5 mM) analysed with BGEs: (a) BGE A : 50 mM phosphoric acid, 25 mM Tris (current 52 *µ*A), (b) BGE B : 50 mM phosphoric acid, 50 mM glycine (current 52 *µ*A), or (c) BGE C : 100 mM phosphoric acid, 100 mM glycine (current 84 *µ*A), at 20 kV, or with (d) BGE D : 100 mM phosphoric acid, 55 mM T-EthA (current 46 *µ*A) at 13 kV. PM: pyridoxamine, B1: thiamine, B3: nicotinamide, PN: pyridoxine, and PL: pyridoxal.

The vitamins pyridoxine (PN), pyridoxal (PL), pyridoxamine (PM), thiamine (B1), and nicotinamide (B3) with pKa values of 5.6, 4.1, 9.6, 5.5, and 3.6, respectively [25], are all positively charged at acidic pH. Therefore, a BGE with a low pH should be used. Phosphate has a pKa value of 2.2; and thus, a high buffering capacity at this pH, which is significantly lower than the pKa values of the vitamins. In addition, it is inorganic, thus showing very little UV absorbance. Hence, phosphoric acid BGEs were investigated. At a low pH, the separation window for cationic substances is wide, which increases the separation of cell culture medium matrix components from the vitamins. One of the attributes a robust method needs to adhere to is good migration time repeatability. Selecting a BGE with a high buffering capacity improves the migration time repeatability [26]. For a robust method, the BGE composition should be consistent. For that reason, the pH of the BGE should be set by using calculated concentrations of buffer components, rather than setting the pH afterwards by the addition of acids or bases. This approach controls ionic strength and reproducibility. In order to ensure sharp peaks, electromigration dispersion should be suppressed by selecting a coion with a mobility close to that of the analytes [27]. The mobilities of pyridoxal, pyridoxine, and nicotinamide were determined to be 26.5, 27.1, and 33.9 × 10^−9^ m^2^/Vs, respectively, by Terekhova et. al. [28], and the mobilities of pyridoxamine and thiamine are expected to be in the same range. Two coions with mobilities close to this range were selected; Tris and glycine. The pKa of phosphoric acid is 2.2; a higher buffering capacity is thus obtained with a BGE pH close to this value. When mixing phosphoric acid and Tris in a ratio of 2 : 1, and phosphoric acid and glycine in a ratio of 1 : 1, a pH of 2.3 is obtained. Three BGEs were prepared; A) 50 mM phosphoric acid, 25 mM Tris, pH 2.3, B) 50 mM phosphoric acid, 50 mM glycine, pH 2.3, and C) 100 mM phosphoric acid, 100 mM glycine, pH 2.3, see Table S1 in the supporting information for buffer properties. A standard vitamin mixture containing vitamin B1, B3, and the B6 complex pyridoxine, pyridoxal, and pyridoxamine was analysed with these three BGEs, see Figures 1(a)–1(c) for the obtained electropherograms and Table S2 in the supporting information for measured mobilities. The migration time repeatabilities of the vitamins achieved with BGE A, B, or C were all within 7.0% RSD, 1.6% RSD, or 0.7% RSD, respectively. As expected, the migration time repeatability increased with increasing buffering capacity. Using glycine in the BGE, unfortunately, caused the vitamin peaks to split into double peaks. The eigen mobility of the BGEs containing glycine is close to the mobilities of the vitamins, which could cause an interference [29]; however, interference caused by the eigen mobility of the buffer is expected to influence only one of the vitamin peaks, not all peaks. Although the mechanism is not fully understood, it is clear that a different coion should be selected. When using Tris in the BGE, not all vitamins were baseline separated. An alternative coion for Tris is T-EthA. T-EthA interacts with the capillary wall, forming a dynamic coating, controlling the EOF, and slightly reversing it [30], which could improve resolution. In addition, the dynamic coating improves robustness as small changes in pH do not affect the EOF as strongly as on an uncoated capillary, it prevents adsorption of cell culture medium components, and it increases migration time reproducibility. For T-EthA, a pH of 2.3 is obtained when mixing phosphoric acid and T-EthA in a ratio of 1.8 : 1. A standard vitamin mixture was analysed with a BGE consisting of 100 mM phosphoric acid and 55 mM T-EthA at pH 2.3 (BGE D). This BGE had a buffering capacity of 69.1 mM, which is about equal to the 50 mM phosphoric acid/50 mM glycine BGE (Table S1). By changing the coion to T-EthA, an improvement in resolution and peak shape was achieved. Due to the high current produced when using this BGE (82 *µ*A at 20 kV), the applied voltage was reduced to prevent excessive Joule heating. Changing the voltage from 20 to 13 kV did not affect the resolution of the vitamin peaks, it only increased the migration times. The migration time repeatabilities for the vitamins using T-EthA in the BGE were all within 0.4% RSD, which is significantly better than when using Tris or glycine as coion.

### 3.2. Application to Cell Culture Medium

**Figure 2:**
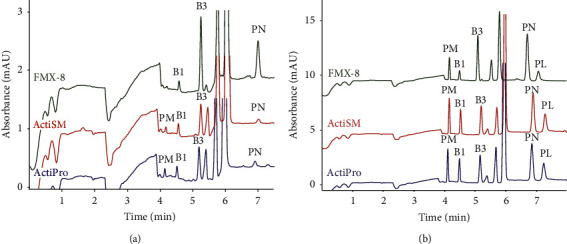
Electropherograms of (a) FMX-8 MOD, ActiSM, and ActiPro medium and (b) FMX-8 MOD, ActiSM, and ActiPro medium spiked with standard vitamins (50 *µ*M) analysed with BGE: 100 mM phosphoric acid, 55 mM T-EthA. Applied voltage 13 kV; resulting current 40–46 *µ*A. PM: pyridoxamine, B1: thiamine, B3: nicotinamide, PN: pyridoxine, PL: pyridoxal.

In order to show the applicability of the developed method for vitamin monitoring in biopharmaceutical processes, three different cell culture media were tested. Two of these media were the commercially available ActiPro medium and ActiSM medium. These cell culture media are commercially available with a proprietary composition but are described as chemically defined and animal-derived component-free media developed to provide high yields of recombinant proteins in bioprocesses using Chinese hamster ovary (CHO) cell lines [32]. The ActiSM medium is generally used for developing the bioprocess, while ActiPro is used for the production process. The third medium was the modified FMX-8 medium (FMX-8 MOD). The FMX-8 culture medium was developed for the production of recombinant proteins under chemically defined culture conditions and its formulation was published [33]. To prepare FMX-8 MOD, several components commonly present in the cell culture medium were added to the well-defined FMX-8 culture medium. The exact composition of FMX-8 MOD was described in [24], FMX-8 MOD contains the cationic vitamins nicotinamide (16.2 *µ*M), thiamine (4.3 *µ*M), and pyridoxine (12.9 *µ*M). The three media were analysed untreated (Figure 2(a)), as well as spiked with a standard vitamin mixture (Figure 2(b)). The vitamins are separated from the components in the complex cell culture medium matrices and detected in the cell culture medium samples. This shows the potential of the method as a platform method to monitor the concentrations of the vitamins in biopharmaceutical processes. A complete validation always needs to be performed with samples from the actual bioprocess under monitoring. The data given here demonstrate that this additional/complementary validation will be straightforward.

Another factor affecting the robustness of the method is capillary preconditioning. To investigate the influence of the preconditioning solvent on the robustness, the capillary was flushed with either 0.1 M phosphoric acid or with 1 M NaOH prior to analysing a standard vitamin mixture six times. The migration time repeatabilities of the vitamins analysed after the phosphoric acid flush were all within 0.8% RSD, while the migration time repeatabilities of the vitamins analysed after the NaOH flush were all within 0.4% RSD. The peak area repeatabilities after the phosphoric acid or NaOH flush were all within 8.2% RSD or 2.6% RSD, respectively. Using a NaOH flush significantly improved both the migration time and the peak area repeatabilities compared to a phosphoric acid flush. It was also observed that the migration times slightly increased after the NaOH flush. The T-EthA forms a dynamic positive coating on the capillary wall, slightly reversing the EOF. After flushing with NaOH, more silanol groups on the capillary wall are deprotonated than after the phosphoric acid flush, which results in more T-EthA adsorbing to the capillary wall, explaining the slightly longer migration times. A trend in decreasing migration times was observed after both preconditioning protocols; however, it was less prominent after the NaOH flush, which indicates that after the NaOH flush a more stable T-EthA coating was formed.

Preliminary method validation was performed for the final method with optimised capillary preconditioning and BGE. Method precision was tested using six consecutive runs of a standard vitamin mixture. The migration time repeatabilities for the vitamins in the mixture were within 0.4% RSD and the repeatabilities of the peak area were all within 2.6% (Table 1). Linearity was investigated over the range of 10–110 *µ*M by triplicate analysis of six standard vitamin mixtures at different concentrations (10 *µ*M, 30 *µ*M, 50 *µ*M, 70 *µ*M, 90 *µ*M, and 110 *µ*M). Calibration plots were linear over this range with *R*^2^ larger than 0.99 for all vitamins (Table 1, and Figure S1, Supporting Information). The lowest level, 10 *µ*M, is the lower range limit QL [31] and was prepared in duplicate and injected in total six times, in order to have good precision and accuracy on the lower range limit of quantification QL. Accuracy was determined as a recovery of the calculated concentrations from the actual concentrations. Overall, the recovery was 95–105%, although the recovery at the lowest concentration level of pyridoxamine was 94% (Table 2). Generally, for vitamin determination in complex cell culture media a recovery of 90–110% and a precision of 10% RSD are required. This method performs well within the acceptable range.

## 4. Conclusion

Summarising the results, the cationic vitamins B1, B3, and the B6 complex pyridoxine, pyridoxal, and pyridoxamine can be separated using a 100 mM phosphoric acid and 55 mM T-EthA BGE at pH 2.3 with good resolution. The dynamic T-EthA coating is most stable after a capillary preconditioning with 1 M NaOH. A good precision was obtained with migration time repeatabilities within 0.4% RSD and peak area repeatabilities within 2.6% RSD. The method was linear over the range of 10–110 *µ*M, with good accuracy of 94–104%. These performance results were well within the required limits of 90–110% accuracy and ≤ 10% RSD. The method shows to be applicable for the monitoring of vitamins in biopharmaceutical processes, the vitamins are separated from the complex cell culture medium matrix. This shows the great potential of using this method for at-line analysis for the monitoring of the biopharmaceutical process. A full validation should be performed with process samples from the actual bioprocess under monitoring. Looking at the preliminary validation data, no issues are expected for the final validation.

## Figures and Tables

**Table 1:** Preliminary method validation data: precision on migration time and peak area of six consecutive runs and linearity data over the range 10–110 *µ*M.

	Precision (*n* = 6)		Linearity (*n* = 21)		
	Migration time (%)	Peak area RSD (%)	Slope	Intercept	Correlation coefficient (*R*^2^)
PM	0.4	1.9	0.11	0.14	0.996
B1	0.4	2.6	0.08	0.23	0.996
B3	0.3	2.3	0.10	0.13	0.998
PN	0.3	1.5	0.25	0.34	0.998
PL	0.3	1.5	0.20	0.24	0.998

**Table 2:** Method accuracy expressed as percentage recovery.

Conc. (*µ*M)	*n*	PM (%)	B1 (%)	B3 (%)	PN (%)	PL (%)
10	6	94	95	95	95	96
30	3	102	101	100	100	98
50	3	102	103	103	103	104
70	3	103	102	102	101	101
90	3	96	97	98	98	100
110	3	101	101	100	100	99

## Data Availability

The data that support the findings of this study are available from Kantisto BV. Restrictions apply to the availability of these data, to allow for the commercialisation of research findings.

## Abbreviations

BGE: Background electrolyte
EOF: Electro-osmotic flow
PL: Pyridoxal
PM: Pyridoxamine
PN: Pyridoxine
T-EthA: Triethanolamine
Tris: Tris(hydroxymethyl)aminomethane

## Supplementary Materials

Supplementary Materials
*Table S1*. BGE composition and properties as calculated by PeakMaster. PA: phosphoric acid, Gly: glycine. The pH is 2.3 for each BGE. *Table S2*. Apparent mobilities (×10^−9^ m^2^/V/s) of the vitamins obtained with the different BGEs. At pH 2.3, the EOF could be assumed virtually zero, making these values also the electrophoretic mobilities. For BGE D, a negative EOF is likely present, decreasing the apparent mobilities of the vitamins. *Figure S1.* Calibration curves (left) and residual plots (right) of (a) pyridoxamine, (b) thiamine, (c) nicotinamide, (d) pyridoxine, and (e) pyridoxal.
