# Supplementary material for: Analysis of Cationic Vitamins in Cell Culture Medium Samples by Capillary Zone Electrophoresis
Source: J Anal Methods Chem. 2022 Oct 6;2022:2819855. doi: 10.1155/2022/2819855 (PMC9560846; doi:10.1155/2022/2819855)
Supplement: Supplementary Materials — Table S1. BGE composition and properties as calculated by PeakMaster. PA: phosphoric acid, Gly: glycine. The pH is 2.3 for each BGE. Table S2. Apparent mobilities (×10−9 m2/V/s) of the vitamins obtained with the different BGEs. At pH 2.3, the EOF could be assumed virtually zero, making these values also the electrophoretic mobilities. For BGE D, a negative EOF is likely present, decreasing the apparent mobilities of the vitamins. Figure S1. Calibration curves (left) and residual plots (right) of (a) pyridoxamine, (b) thiamine, (c) nicotinamide, (d) pyridoxine, and (e) pyridoxal. [file 2819855.f1.zip › Supporting Information (1).docx]

**Supplementary data**

**Analysis of cationic vitamins in cell culture medium samples by capillary zone electrophoresis**

Debbie van der Burg^1,2^, Hermann Wätzig^2^, Cari E Sänger – van de Griend^1^

1. Kantisto BV, Callenburglaan 22, 3742 MV Baarn, the Netherlands
2. Institute of Medicinal and Pharmaceutical Chemistry, TU Braunschweig, Braunschweig, Germany

**Keywords:** Vitamins, cell culture medium, capillary zone electrophoresis

**Abbreviations: BGE**, background electrolyte; **EOF**, electro-osmotic flow;
**PL**, pyridoxal; **PM**, pyridoxamine; **PN**, pyridoxine; **T-EthA**, triethanolamine;
**Tris**, tris(hydroxymethyl)aminomethane

**Table S1.** BGE composition and properties as calculated by Peakmaster^[[1]](#footnote-1)^. PA: phosphoric acid, Gly: glycine. The pH is 2.3 for each BGE.

| **BGE** | **Ionic strength (mM)** | **Conduc-tivity**  **(S/m)** | **Buffering capacity (mM)** | **System eigen-mobility** | **Co-ion mobility**  **(x10^-9^ m^2^/V/s)** |
| --- | --- | --- | --- | --- | --- |
| A: 50 mM PA, 25 mM Tris | 30.9 | 0.34 | 40.5 | 49.5 | 24.4 |
| B: 50 mM PA, 50 mM Gly | 31.9 | 0.35 | 68.2 | 18.3 | 18.0 |
| C: 100 mM PA, 100 mM Gly | 62.5 | 0.54 | 125.4 | 14.6 | 18.2 |
| D: 100 mM PA, 55 mM T-EthA | 61.4 | 0.49 | 69.1 | 41.7 | 23.8 |

**Table S2.** Apparent mobilities (x10^-9^ m^2^/V/s) of the vitamins obtained with the different BGEs. At pH 2.3, the EOF could be assumed virtually zero, making these values also the electrophoretic mobilities. For BGE D, a negative EOF is likely present, decreasing the apparent mobilities of the vitamins.

| **BGE** | **B1** | **B3** | **PL** | **PM** | **PN** |
| --- | --- | --- | --- | --- | --- |
| A: 50 mM PA, 25 mM Tris | 32.3 | 28.9 | 24.3 | 35.0 | 25.0 |
| B: 50 mM PA, 50 mM Gly | 28.1 | 23.5 | 18.6 | 36.3 | 19.0 |
| C: 100 mM PA, 100 mM Gly | 29.4 | 24.8 | 20.0 | 36.0 | 23.6 |
| D: 100 mM PA, 55 mM T-EthA | 22.2 | 19.4 | 13.3 | 25.1 | 14.1 |

**Figure S1.** Calibration curves (left) and residual plots (right) of A) pyridoxamine, B) thiamine, C) nicotinamide, D) pyridoxine, and E) pyridoxal.

1. i https://web.natur.cuni.cz/gas/peakmaster.html [↑](#footnote-ref-1)
