# Supplementary material for: Analysis of Cationic Vitamins in Cell Culture Medium Samples by Capillary Zone Electrophoresis
Source: J Anal Methods Chem. 2022 Oct 6;2022:2819855. doi: 10.1155/2022/2819855 (PMC9560846; doi:10.1155/2022/2819855)
Supplement: Supplementary Materials — Table S1. BGE composition and properties as calculated by PeakMaster. PA: phosphoric acid, Gly: glycine. The pH is 2.3 for each BGE. Table S2. Apparent mobilities (×10−9 m2/V/s) of the vitamins obtained with the different BGEs. At pH 2.3, the EOF could be assumed virtually zero, making these values also the electrophoretic mobilities. For BGE D, a negative EOF is likely present, decreasing the apparent mobilities of the vitamins. Figure S1. Calibration curves (left) and residual plots (right) of (a) pyridoxamine, (b) thiamine, (c) nicotinamide, (d) pyridoxine, and (e) pyridoxal. [file 2819855.f1.zip › Supporting Information.pdf]

Supplementary data

Analysis of cationic vitamins in cell culture medium samples by capillary zone electrophoresis

Debbie van der Burg[1,2], Hermann Wätzig[2], Cari E Sänger – van de Griend[1]

[1] Kantisto BV, Callenburglaan 22, 3742 MV Baarn, the Netherlands

[2] Institute of Medicinal and Pharmaceutical Chemistry, TU Braunschweig, Braunschweig, Germany

Keywords: Vitamins, cell culture medium, capillary zone electrophoresis

Abbreviations: **BGE**, background electrolyte; **EOF**, electro-osmotic flow; **PL**, pyridoxal; **PM**, pyridoxamine; **PN**, pyridoxine; **T-EthA**, triethanolamine; **Tris**, tris(hydroxymethyl)aminomethane

Table S1. BGE composition and properties as calculated by Peakmaster[i]. PA: phosphoric acid, Gly: glycine. The pH is 2.3 for each BGE.

BGE	Ionic strength (mM)	Conduc-tivity (S/m)	Buffering capacity (mM)	System eigen-mobility	Co-ion mobility (x10^{-9} m^2/V/s)
A: 50 mM PA, 25 mM Tris	30.9	0.34	40.5	49.5	24.4
B: 50 mM PA, 50 mM Gly	31.9	0.35	68.2	18.3	18.0
C: 100 mM PA, 100 mM Gly	62.5	0.54	125.4	14.6	18.2
D: 100 mM PA, 55 mM T-EthA	61.4	0.49	69.1	41.7	23.8

Table S2. Apparent mobilities (x10^{-9} m^2/V/s) of the vitamins obtained with the different BGEs. At pH 2.3, the EOF could be assumed virtually zero, making these values also the electrophoretic mobilities. For BGE D, a negative EOF is likely present, decreasing the apparent mobilities of the vitamins.

BGE	B1	B3	PL	PM	PN
A: 50 mM PA, 25 mM Tris	32.3	28.9	24.3	35.0	25.0
B: 50 mM PA, 50 mM Gly	28.1	23.5	18.6	36.3	19.0
C: 100 mM PA, 100 mM Gly	29.4	24.8	20.0	36.0	23.6
D: 100 mM PA, 55 mM T-EthA	22.2	19.4	13.3	25.1	14.1

[i] https://web.natur.cuni.cz/gas/peakmaster.html

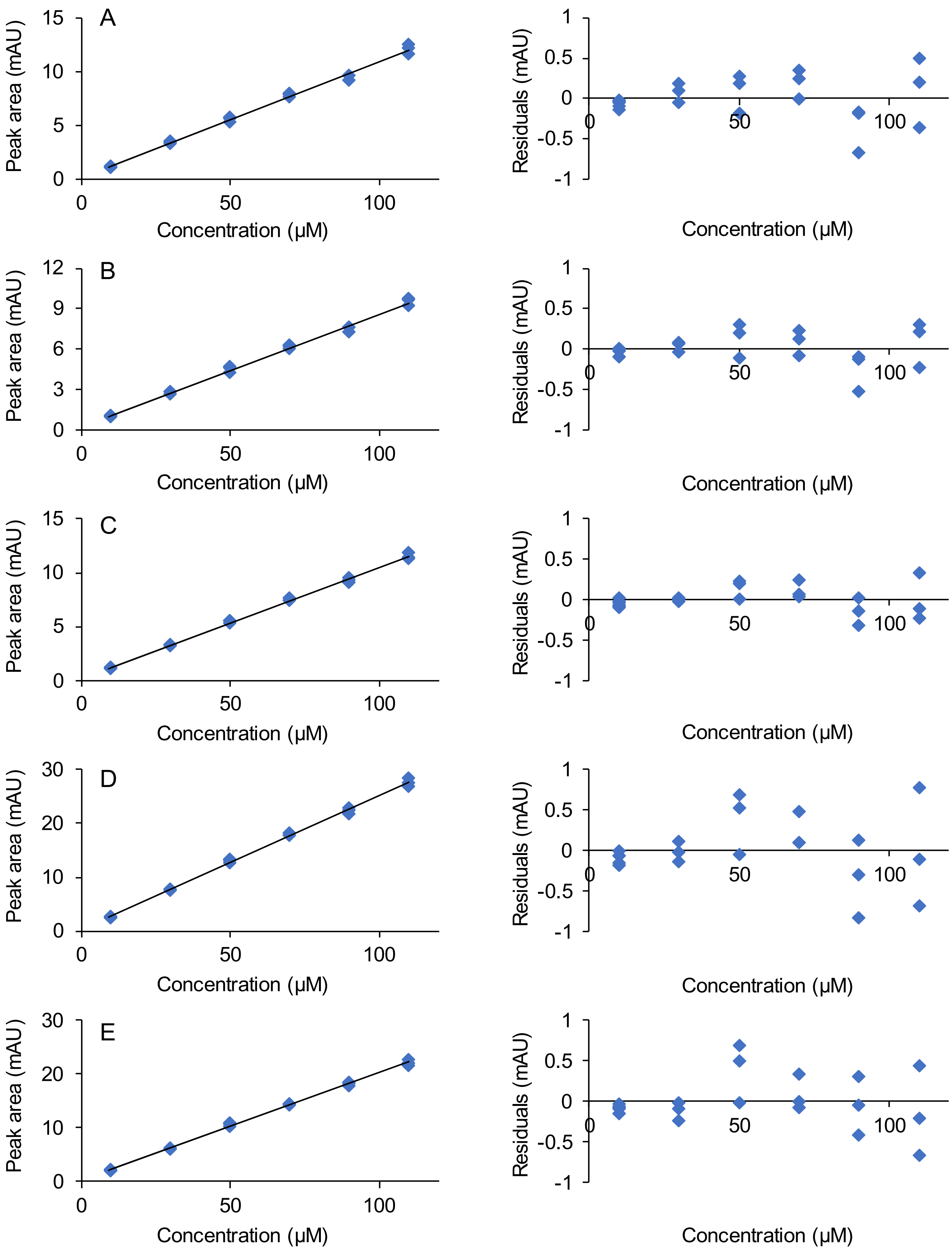

Figure S1. Calibration curves (left) and residual plots (right) of A) pyridoxamine, B) thiamine, C) nicotinamide, D) pyridoxine, and E) pyridoxal.
